# A Misleading Trismus

**DOI:** 10.1155/2011/969621

**Published:** 2011-07-20

**Authors:** A. Galzerano, E. Sabatini, A. De Monte, D. Durì

**Affiliations:** ^1^Unità Area Critica di Terapia Intensiva (A.C.U.T.I.), Azienda Ospedaliero-Universitaria S. Maria della Misericordia di Perugia, S. Andrea delle Fratte, 06156 Perugia, Italy; ^2^Anestesia e Rianimazione 1 Servizio, Azienda Ospedaliero-Universitaria S. Maria della Misericordia, Piazzale S. Maria della Misericordia, 33100 Udine, Italy

## Abstract

We report the case of a 73-year-old women admitted to ICU with a diagnosis of tetanum supported by a mild trismus and treated with potentially unsafe drugs until the correct diagnosis was made.

## 1. Introduction

Acute intermittent porphyria is a disease common enough to be encountered by many physicians but rare enough to lead to a wrong diagnosis.

## 2. Report of the Case

A 73-year-old male patient came to our attention after admission to ICU from infective disease ward with the diagnosis of tetanus. His past clinical history was significant for recent use of a nonreported antibiotic for a sinusitis and a recent injury on his left hand with a garden tool. At the admission the patient was conscious with intense abdominal pain, blood pressure was 210/110 mmHg, 100 bpm, Sp02 = 89% in air, red urine output due to difficult bladder catheterization, and mild trismus. Abdomen was soft, with mild tenderness. The patient began specific antibiotic treatment, immunoglobulins, and benzodiazepines (diazepam) to treat muscle rigidity, diclofenac, and metoclopramide for abdominal pain and vomiting with no significant clinical improvement. Tetanus antibodies resulted negative. After admission, abdominal pain worsened with no positive radiological examinations (abdominal TC and echography) and bleeding from catheter also continued in spite of frequent bladder washing with continuous cold saline. On the base of abdominal pain and red urine output, acute intermittent porphyria (AIP) was suspected and the qualitative test for urinary porphyrins was positive. The patient was given dextrose at rate of 200 gr/24 h, and the day after abdominal pain resolved and the urine colour became clearer. The patient never required intubation as hypoxemia responded to oxygen supplementation through facemask, and neurological status never deteriorated, so the trismus did not interfere with airway patency. 

The patient was then readmitted to infective disease ward for genetic diagnosis.

## 3. Discussion 

Many drugs used during recovery were useful in the hypothesis of tetanus but not safe (metoclopramide) or contraindicated (diazepam and diclofenac) in AIP and may have worsened the attack, which was probably induced by the unspecified antibiotic used to treat sinusitis. Red urine output should have risen before the suspicion of AIP, but very difficult catheterization was misleading, until associated with nonimproving abdominal pain, but the most misleading factor was the previous diagnosis of tetanus on the basis of anamnesis and mild trismus only, though abdomen was soft, with no muscular spasm. The presence of trismus contrasts the usual weakness or paralysis typical of AIP, and in this case remains unexplained. Differential diagnosis of trismus includes malignant neuroleptic syndrome, but in this case no neuroleptic was administered before or after admission to intensive care unit. We enhance then the importance of performing early urinary test for porphyrins in the presence of abdominal pain and red urine output.

